# Informational Structures and Informational Fields as a Prototype for the Description of Postulates of the Integrated Information Theory

**DOI:** 10.3390/e21050493

**Published:** 2019-05-14

**Authors:** Piotr Kalita, José A. Langa, Fernando Soler-Toscano

**Affiliations:** 1 Faculty of Mathematics and Computer Science, Jagiellonian University, ul. Łojasiewicza 6, 30-348 Kraków, Poland; 2 Department of Differential Equations and Numerical Analysis, Faculty of Mathematics, University of Seville, 41080 Seville, Spain; 3 Department of Philosophy, Logic and Philosophy of Science, Faculty of Philosophy, University of Seville, 41018 Seville, Spain

**Keywords:** dynamical system, integrated information theory, global attractors, Lotka–Volterra equations, informational structure, informational field

## Abstract

Informational Structures (IS) and Informational Fields (IF) have been recently introduced to deal with a continuous dynamical systems-based approach to Integrated Information Theory (IIT). IS and IF contain all the geometrical and topological constraints in the phase space. This allows one to characterize all the past and future dynamical scenarios for a system in any particular state. In this paper, we develop further steps in this direction, describing a proper continuous framework for an abstract formulation, which could serve as a prototype of the IIT postulates.

## 1. Introduction

The dynamics of many real phenomena can be described by systems of difference or differential equations on structural complex networks. A system of nonlinear differential equations (sometimes including noise, delays, or time-dependent coefficients) is frequently used to describe the global interrelated activity on nodes that are mutually connected, with those connections represented by an adjacency matrix. In this way, global dynamics emerges from the dynamics on each node, coupled to others as described in the associated structural network. Thus, the real phenomena are described by graphs together with associated dynamics, which allows us to define a continuous flow of data on structural networks. Global brain dynamics is one of the promising areas following this general perspective where it is possible to understand the function from the analysis of the time behavior of real measured data on a particular parceling (structural network) of the brain [1,2,3,4,5]. The study of patterns (such as correlation, synchronization, or metastability) on these dynamical processes has received much attention in the last few years [6,7]. Most of these papers analyzed the functional networks associated with the dynamics on structural networks. In this paper, we take advantage of some of the results in the important theory of dynamical systems related to the characterization of the global attractor, which can be seen as an invariant structure (or attracting complex network [8,9]) determining all the possible past and future scenarios of a system in any particular state.

Integrated Information Theory (IIT) [10] develops a theory of consciousness starting from the phenomenological axioms satisfied by any conscious experience. Essentially, the theory states that a mechanism in a particular state is conscious when it produces intrinsic integrated information, which satisfies all the postulates of the theory.

**Figure 1:**
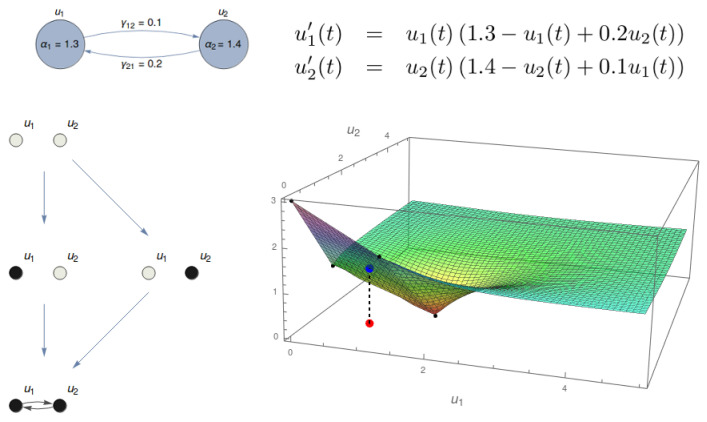
The dynamics on a substrate with two nodes (top left) produces an Informational Structure (IS) and Informational Field (IF). In the example, $\alpha_{1}=1.3$, $\alpha_{2}=1.4$, $\gamma_{12}=0.1$, $\gamma_{21}=0.2$. The dynamics of the substrate is given by a system of differential equations (top right). The IS (bottom left) contains the stationary points and relations among them. The IF (bottom right) enriches the phase space with the Lyapunov functional. In the example, the point (0.8,0.9) in the phase space (red point) is associated with a certain level in the IF (blue point).

In [11], we proposed a continuous treatment of IIT notions. We start by considering a substrate regulated by a system of differential equations (top of Figure 1). The dynamics of the system is characterized by its associated Informational Structure (IS), containing all stationary points and relations among them (Figure 1, bottom left). Our approach to IIT in [11] started at the IS and used it to define Transition Probability Matrices (TPMs) with the probability distributions to the future and to the past for any point in the IS. Using the TPMs, we proceeded in an analogous way to IIT [10]. In this paper, we go one step further in the direction of defining IIT notions in a continuous dynamical framework. Instead of creating TPMs, we look at the Informational Field (IF; see Figure 1, bottom right), which is defined by a Lyapunov functional that enriches all the points in the phase space with information. Any point in the phase space that belongs to the global attractor (for example, the red point in the IF of Figure 1) is associated with a point in the IF (blue point) that has access both to the past and to the future of a specific portion of the IF. The IIT key notions will be defined in pure dynamical terms by using the system of differential equations and associated IF, which is the base to measure the cause and effect information. An important remark is that the dynamics of any actual state can be calculated by looking at the system of equations and considering the trajectories of that state as t↦−∞ and t↦+∞. For any particular point in the global attractor of the system, which is our phase space, these trajectories tend toward specific points (or, more generally, minimal invariant sets) in the IS. This could be considered as the analogy of past and future scenarios in IIT described by a TPM.

## 2. Integrated Information Theory of Consciousness

We devote this section to a brief presentation of the fundamental notions of the Integrated Information Theory (IIT) [10]. The starting point of IIT is a phenomenological characterization of the conscious experience through five axioms:

- Existence: Conscious experience has an intrinsic existence. It is the only thing whose existence is evident.
- Composition: Consciousness is structured by multiple aspects. For example, the same experience can be composed both by auditory and visual aspects.
- Information: Each conscious experience is specific, it is only one of the many possible experiences that is presented in a particular way different from the rest of the possibilities.
- Integration: Consciousness is unified. One is not separately conscious of the auditory and visual experiences; both stimuli are integrated in the same conscious experience.
- Exclusion: Each experience excludes all others and has a particular spatial and temporal scale. It also has definite borders; a subject may not be conscious of all the stimuli he/she is receiving.

Once the axioms have been presented, IIT introduces the postulates that have to be satisfied by any physical system having conscious experiences. Each postulate is related to an axiom. We now discuss these details of IIT postulates for mechanisms that are relevant for the comparison with our proposal. For a detailed description, see the glossary in [10]. The existence postulate requires that a mechanism (typical examples of IIT are based on logical circuits with several nodes) in a particular state (nodes of the circuit have a specific value of zero or one) has cause-effect power. Intrinsic information of a state is defined by its cause (the past) and effect (the future) power. This is then represented by a Transition Probability Matrix (TPM), which specifies the probability of moving from any particular state of the system to any other. The composition postulate states that elementary mechanisms (logic gates, for example) can be combined to form higher order mechanisms (logic circuits). The information postulate measures the information of a system in a particular state. Information is measured by comparing the cause and effect repertoires (the selection produced by the state) on the unconstrained past and future. Given the actual state of the system, its cause repertoire is the probability distribution of possible past states given the current state (computed from the TPM). The unconstrained past is the uniform probability distribution of all possible past states of the system. The distance between both distributions (the Earth Mover’s Distance (EMD) is used in [10]) is the cause-information (ci). The effect-information (ei) is defined in a similar way by considering probability distributions to the future (there are some particularities in the way that the unconstrained future is defined). Then, the cause-effect information (cei) is the minimum of ci and ei. This way, a state of the system is informative when it makes a difference with respect to not knowing the state, both for the past and the future. The approach to measure information proposed in this paper is based on calculating ci and ei directly from the IS and IF. We stress here that to measure information, IIT considers a candidate set and not only the whole system, but for simplicity in this paper, we only show how to calculate information for the whole system; hence, our proposal to measure the information is not based on the partition and composition. As a consequence, we do not show here a compositional way of measuring information as in IIT 3.0. However, we think it would be possible to generalize the proposed approach to calculate information in a compositional way in analogy to IIT 3.0. Indeed, given a substrate in a given state, we could also measure information for the past and future scenarios for all possible partitions of the candidate set and then proceed as IIT does. In any case, in our treatment, we are still only calculating (small) φ and not (big) Φ, which is also an important difference, in particular for the discussion of the exclusion postulate.

The integration postulate quantifies how much information of the whole cannot be obtained from its parts. To do that, all partitions of the system are considered. Partitions are obtained by dividing the system into two parts and noising the connections between the parts. The distance (EMD) between the cause repertoire of the system in the current state and the closest cause repertoire of all partitions (Minimum Information Partition (MIP)) is $\varphi_{\mathrm{cause}}$. The calculation of $\varphi_{\mathrm{effect}}$ is analogous. Finally, the integration φ is the minimum of $\varphi_{\mathrm{cause}}$ and $\varphi_{\mathrm{effect}}$. Finally, the exclusion postulate chooses the submechanism of the given system for which the cause-effect repertoire has the highest integration value, $\varphi^{\mathrm{Max}}$.

## 3. Materials and Methods

In this section, we present the main mathematical notions from [11] that are used in Section 4 to interpret IIT notions from a dynamical systems perspective. The main concepts are the Informational Structure (IS) and Informational Field (IF). For a more detailed presentation, see our previous paper [11].

### 3.1. The Global Attractor

The mathematical way of describing nonlinear dynamics in our case is governed by differential equations. In particular, we consider systems of ordinary differential equations given by:

(1) $$\left\{\begin{array}{c} \frac{du}{dt}=F\left(u\right)\\ u\left(t_{0}\right)=u_{0} \end{array}\right.$$

with the nonlinearities represented by *F*, a map from $R^{N}$ to $R^{N}$. The initial datum $u_{0}$ is the state at time $t_{0}$.

Given the phase space X, define the family of non-linear operators {S(t):t≥0} as:

S(t):X→X,u∈X,S(t)u∈X

which defines the dynamics of any element u∈X. The mapping $t\mapsto S\left(t\right)u_{0}=u\left(t;u_{0}\right)$ is the solution of (1) as a function of time *t* with the initial datum equal to $u_{0}$ taken at time $t_{0}=0$.

The main concept to describe the future scenarios of a system is the global attractor ([12,13,14,15,16]):

**Definition** **1.** *A set* A⊆X *is a global attractor for* {S(t):t≥0} *if it is:*

*(i)*  *compact,*
*(ii)*  *invariant under* {S(t):t≥0} *, i.e.,* S(t)A=A *for all* t≥0, *and*
*(iii)*  *attracts bounded subsets of X under* {S(t):t≥0} *; that is, for all* B⊂X *bounded:*
  $${\mathrm{dist}}_{X}\left(S\left(t\right)B,A\right)\overset{t\to \infty }{⟶}0.$$

Note that for nonempty sets A,B⊂X, by ${\mathrm{dist}}_{X}\left(B,A\right)$, we mean the Hausdorff semidistance between the two sets defined as ${\mathrm{dist}}_{X}\left(B,A\right)=\sup_{b\in B}\inf_{a\in A}∥a-b∥$.

Through every point in A, there passes the global trajectory u(t) defined and contained in A for every t∈R. Essentially, for our goal of the interpretation of IIT, we restrict to the case X=A, so that every point on the *X* can watch, or possesses all the information, about all its future (effect power) and its past (cause power).

A state $u^{*}\in X$ is an equilibrium point for the semigroup S(t) if $S\left(t\right)u^{*}=u^{*},$ for all t≥0. Any invariant set is a subset of the global attractor [13]. Connections among invariant sets in the attractor describe its structure [12,17,18].

Our approach to IIT will be based on the Informational Structures (Section 3.3) and Informational Fields (Section 3.4) associated with a global attractor. Typically, these structures possess nodes given as stationary points, periodic orbits [19,20], or minimal invariant sets with chaotic dynamics [21,22]. We can conceive of a global attractor as an object of informational nature. It is composed by a set of special solutions, connecting particular invariants, so describing a complex directed graph [23].

The following concept of the Lyapunov functional associated with an attractor for which its fine structure is known is crucial, as it describes the way in which the phase space is curved in order to define the cause and effect power of every state of the system. This is one of the crucial points in order to define the intrinsic informational contents of a state.

**Definition** **2** ([13,17,24])**.** *We say that a semigroup* {S(t):t≥0} *with a global attractor* A *and a disjoint family of isolated invariant sets* $E=\{E_{1},\cdots ,E_{n}\}$*,* $E_{i}\subset X$ *for all* i=1,⋯,n, *is a gradient semigroup with respect to* E *if there exists a continuous function* V:X→R *such that:*

*(i)*  [0,∞)∋t↦V(S(t)x)∈R*is non-increasing for each*x∈X*;*
*(ii)*  *V is constant in*$E_{i}$*, for each*1≤i≤n*; and*
*(iii)*  *if*V(S(t)x)=V(x)*for all*t≥0*, then*$x\in \overset{n}{\underset{i=1}{⋃}}E_{i}$*.*

*In this case, we call V a Lyapunov functional related to* E *.*

Note that a Lyapunov functional is defined in *X* with values in R, so that it describes a scalar deformation of the phase space X. In particular, it is constant on each set $E_{i}$ and strictly decreasing outside these invariants. In this way, a Lyapunov functional pictures a landscape based on the family of sets $E_{i}$ and connecting surfaces, of different dimensions, among them.

We recall the notion of stable and unstable sets of the set Ξ⊂X. In particular, the notion of the unstable set is crucial in order to characterize attractors for gradient systems.

$$\begin{array}{l} W^{u}\left(\Xi \right)=\{z\in X:\mathrm{there}\;\mathrm{is}\;a\;\mathrm{global}\;\mathrm{solution}\xi :R\to X\mathrm{for}S\left(t\right)\\ \;\;\;\mathrm{satisfying}\xi \left(0\right)=z\mathrm{and}\;\mathrm{such}\;\mathrm{that}\lim_{t\to -\infty }{\mathrm{dist}}_{X}\left(\xi \left(t\right),\Xi \right)=0\}.\\ W^{s}\left(\Xi \right)=\{z\in X:\mathrm{there}\;\mathrm{is}\;a\;\mathrm{global}\;\mathrm{solution}\xi :R\to X\mathrm{for}S\left(t\right)\\ \;\;\;\mathrm{satisfying}\xi \left(0\right)=z\mathrm{and}\;\mathrm{such}\;\mathrm{that}\lim_{t\to \infty }{\mathrm{dist}}_{X}\left(\xi \left(t\right),\Xi \right)=0\}. \end{array}$$

The structure of the global attractor of gradient nonlinear semigroups is described as follows:

**Theorem** **1** ([13,18])**.** *Let* {S(t):t≥0} *be a gradient semigroup with respect to the finite set* $E:=\{E_{1},E_{2},\cdots ,E_{n}\}$*, which has a global attractor* A*. Then,* A *can be written as the union of the unstable sets related to each set in* E*, i.e.,*

(2) $$A=\overset{n}{\underset{j=1}{⋃}}W^{u}\left(E_{j}\right).$$

The above description of a gradient system defines a Morse decomposition for the global attractor [17,19,20,23,25,26].

Thus, given any state *x* in the global attractor, there exists a complete solution through *x*, i.e., ξ:R→X such that ξ(t+s)=S(t)ξ(s) for all t≥0,s∈R with ξ(0)=x. Moreover, it satisfies that for every x∈A, either $x\in E_{i}$ for some *i* or there exist $E_{j},E_{i}\in E$ such that:

(3) $$\lim_{t\to -\infty }{\mathrm{dist}}_{X}\left(S\left(t\right)x,E_{j}\right)=0=\lim_{t\to +\infty }{\mathrm{dist}}_{X}\left(S\left(t\right)x,E_{i}\right),\;with\;j<i.$$

The attractor then gives rise to a new complex dynamical network for all the possible feasible future scenarios and, inside it, also all the possible past ones [8,9].

For a better description of the (informational) landscape drawn by gradient systems, note that any Morse decomposition $E=\{E_{1},\cdots ,E_{n}\}$ of A, thanks to (3), leads to a partial order among the isolated invariant sets $E_{i}$; that is, two invariant sets $E_{i}$ and $E_{j}$ are in relation if there exists a chain of complete solutions:

(4) $$\left\{\xi_{\ell },\;1\leq \ell \leq r-1\right\}\;\mathrm{with}\;\lim_{t\to -\infty }{\mathrm{dist}}_{X}\left(\xi_{\ell }\left(t\right),E_{\ell }\right)=0\;\mathrm{and}\;\lim_{t\to \infty }{\mathrm{dist}}_{X}\left(\xi_{\ell }\left(t\right),E_{\ell +1}\right)=0$$

for 1≤ℓ≤r−1, with $E_{1}=E_{i}$ and $E_{r}=E_{j}$. From [27], we know that there exists a Morse decomposition given by the energy levels $N=\{N_{1},N_{2},\cdots ,N_{p}\}$, p≤n. The associated Lyapunov functional has decreasing values in any two different level sets of N, and any two different elements of E in the same level are not in relation.

### 3.2. An N-Dimensional Lotka–Volterra Cooperative Model

Systems of differential equations of the Lotka–Volterra type have been used to describe transient and asymptotic behavior in neural networks [5,28,29,30,31], trying to understand some specifics of brain activity. For a general situation with *N* nodes, define a system of *N* differential equations written as:

(5) $$\frac{du_{i}}{dt}=u_{i}\left(\alpha_{i}+\sum_{j=1}^{N}\gamma_{ij}u_{j}\right),\;\;\;\;i=1,\ldots ,N,$$

with $A=\left(\gamma_{ij}\right)\in R^{N\times N}$ the matrix describing connections. In matrix formulation, we can write (5) as:

(6) $$\frac{du}{dt}=u^{⊤}\cdot \left(\alpha +Au\right),\;\;\;\;$$

with $A\in R^{N\times N}$ and $\alpha \in R^{N}$ given by $\alpha =(\alpha_{1},\ldots ,\alpha_{N})$. *A* represents the adjacency matrix for a given substrate, which may be related to a structural network associated with a physical brain. We will use (6) as a prototype model to illustrate the dynamics on graphs, which, due to its gradient nature, will be very powerful in order to develop our approach to IIT postulates.

Given initial data for (6), the existence and uniqueness of solutions is well known [32]. For the system (6), the orthant of positive values:

$$R_{+}^{N}=\left\{u=\left(u_{1},\ldots ,u_{N}\right)\in R^{N},u_{i}\geq 0,\;i=1,\ldots ,N\right\}$$

is positively invariant, and thus, the system defines a semigroup of nonlinear operators on this orthant. Under appropriate assumptions on A,α, this system is a gradient and has a global attractor [32].

Lotka–Volterra systems constitute a nonlinear and nontrivial class of examples where the characterization of attractors is, to some extent, well understood [8,9,33]. Under some conditions, we know that there exists a finite number of equilibria and directed connections building a gradient hierarchical organization by level sets of equilibria [9]. The global attractor is uniquely defined for any given set of parameters in (6) and is the starting point for our definition of an informational structure.

### 3.3. Informational Structures

The informational nature of a global attractor is related to the fact that its existence does not emerge from empirical measurements. Its existence has to be understood in an intrinsic way to the system, associated with a substrate, as a set of selected solutions forming a complex structure with the power to determine the behavior of all solutions.

**Figure 2:**
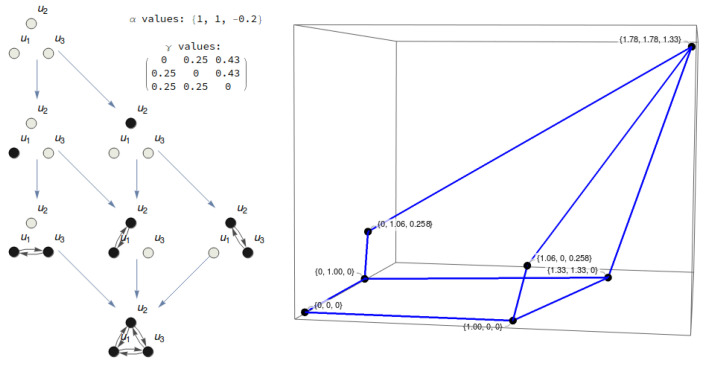
Informational structure of a three-node substrate with the given values of parameters α and γ. In the right figure, the stationary points are shown in the phase space. The blue lines represent solutions joining two stationary points, which belong to different energy levels. Two points are linked if there exists a complete solution converging to the past towards the first one and towards the other point for the future.

We now define an informational structure (Figure 2) for our model (5). Let G be a structural graph with *N* nodes and edges among them. Denote by $G_{i}$ a subgraph of G.

**Definition** **3.** *An Informational Structure (IS) for* (5) *is a complex graph* (I,V)*, with* $I=\{G_{1},\ldots ,G_{M}\}$*, the vertices of which are subgraphs* $G_{i}$ *of* G*, and* V *is the set of directed edges. The vertices* $G_{i}$ *are defined by the equilibria (more generally, minimal invariant sets) of the system of ODEs and edges by the existence of heteroclinic connections between them.*

We may want the IS to flow in time. Then, we consider the system of differential equations driven by time-dependent sources α(t) (and/or $\gamma_{ji}\left(t\right)$), given by:

(7) $${\dot{u}}_{i}=u_{i}\left(\alpha_{i}\left(t\right)+\sum_{j=1}^{N}\gamma_{ij}\left(t\right)u_{j}\right),\;\;\;\;i=1,\ldots ,N.$$

**Figure 3:**
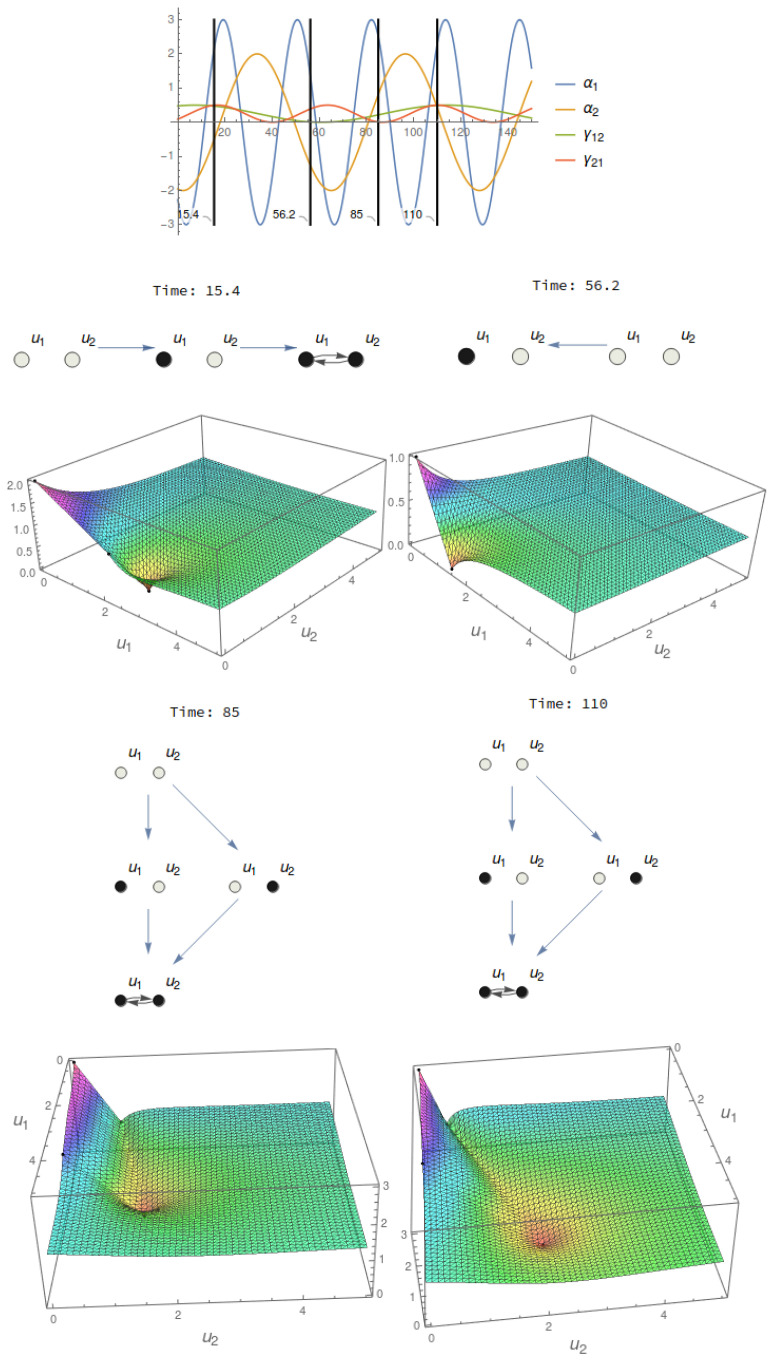
Dynamical IS and IF. Top: Temporal evolution of the parameters α and γ of a two-node mechanism. Bottom: ISs and IFs at four different instants. Observe that the third and fourth have the same structure, but given that the parameters are different, the shape of the IF changes. This fact will affect the informational measures of a state in the phase space.

We do not consider the above system as a non-autonomous one, but rather, a family of autonomous systems parameterized by *t*. Then, for each time t∈R, $\alpha \left(t\right),\gamma_{ij}\left(t\right)$ determine an informational structure I(t), which allows us to define a continuous change on ISs, becoming a dynamical informational structure (I(t),V(t)) (see Figure 3).

### 3.4. Informational Fields

The Informational Field (IF) is a continuous function that maps the phase space $R_{+}^{N}$ to R (see Figure 3). Although IF is defined on the whole $R_{+}^{N}$, we will consider its restriction to A, because only points from A have a well-defined past. Any Lyapunov functional, which always exists for gradient-like semigroups, is a possible IF. Note, however, that in practice, it is usually difficult to construct a Lyapunov functional.

**Lemma** **1** ([17])**.** *For every gradient-like semigroup, there exists a Lyapunov functional. The possible Lyapunov functional is given by:*

$$V\left(x\right)=\sum_{k=1}^{n}\underset{t\geq 0}{\sup}\frac{d(S\left(t\right)x,A_{j})}{d\left(S\left(t\right)x,A_{j}\right)+d\left(S\left(t\right)x,A_{j}^{*}\right)},$$

*where* $A_{j}$ *and* $A_{j}^{*}$ *are the so-called local attractors and repellers associated with the Morse decomposition* E*, respectively.*

As an example, if the Morse decomposition consists of singletons (equilibria of the system), then the item (ii) of Definition 2 is always satisfied. Item (iii) states that *V* is nonconstant along trajectories that are not equilibria of the system. Together with (i), (iii) implies that if for a complete trajectory ξ:R→X, there holds $\lim_{t\to -\infty }u\left(t\right)=x_{i}$ and $\lim_{t\to \infty }u\left(t\right)=x_{j}$, then $V\left(x_{j}\right)<V\left(x_{i}\right)$.

The IS is just the structure supporting an entire informational landscape as a result of a global curvature of the phase space. This is produced by the Lyapunov functional associated with the gradient dynamical system. Given a Lyapunov functional V:X→R associated with the family of isolated invariant sets $E=\{E_{1},\cdots ,E_{n}\}\subset A$, we call the Informational Field (IF) associated with (X,V,E) to the set X={(x,V(x)):x∈A}. The reason we consider the IF restricted to A is because all points in A are the only ones in *X* possessing a well-defined and bounded past for all times going to −∞.

The dynamics of System (6) possesses a global attractor [9], which in this case is a structured set of (at most) $2^{N}$ stationary points (or equilibria) of the system and the trajectories that connect them. Each equilibrium can be uniquely represented by its set of nonzero variables. One of the equilibria is always a globally-stable stationary point [32]. There is a hierarchy made by levels, represented by horizontal rows in the IS, with just one minimalelement corresponding to the globally-stable point. Edges represent complete solutions joining different points. Moreover, there exists a Lyapunov functional *V* associated with the attractor, so that the attractor in this case is organized by informational levels ${\left\{L_{k}\right\}}_{k=0}^{r}$, each level being defined by the stationary points ${\left\{u_{p}^{*,k}\right\}}_{p=1}^{m(k)}$ with the same *V*-value for all $u_{p}^{*,k}\in L_{k}$. In particular, we can take $V(u_{p}^{*,k})=r-k$ for all $u_{p}^{*,k}\in L_{k}$ so that the globally-stable point has the value of *V* equal to zero and the least stable point has value *r*. No connections are possible between the points on the same level having the same value *k*. Moreover, there exists at least one global solution connecting every point in level $L_{k}$ with a stationary point in $L_{k-1}$ as t→−∞ and at least one connecting with a stationary point in $L_{k+1}$ as t→+∞. There exists a connection between $u^{*}\in L_{k}$ and $v^{*}\in L_{q}$ if the set of non-zero values in $u^{*}$ is a subset of non-zero values in $v^{*}$.

In essence, an informational field associated with (6) draws an *N*-dimensional landscape with a higher point at the globally-unstable stationary (zero) solution at level $L_{0}$, which descends through all the semi-stable equilibria to end, at most, at the global stable stationary solution. When all the levels from zero to *N* are present, each level increases in one unit the dimension of the stable manifolds of its stationary points, so that the dimension of the stable manifolds of points $u^{*}\in L_{k}$ is equal to *k*, and hence, the dimension of their associated unstable manifolds is N−k.

The IF enriches every point *u* in the global attractor with a level of information. The length of the global solution passing through *u*, in essence, is related with the number of levels the solution has to cross to reach its asymptotic behavior (past behavior, when t→−∞, related to its cause power, and future behavior, when t→+∞, associated with its effect power).

Every stationary point in the IS possesses an amount of information (as expressed in [11]). However, moreover, every point of the phase space is enriched by the information from the IF. Indeed, the dynamics of every particular realization on the former initial structural graph (the substrate) is determined by the IF. This is why, related to a conscious experience, ISs and associated IFs seem to be promising objects to be studied [34]. Actually, it is the IF that is really showing the cause-effect power of an IS (Figure 3).

### 3.5. Metastability

**Figure 4:**
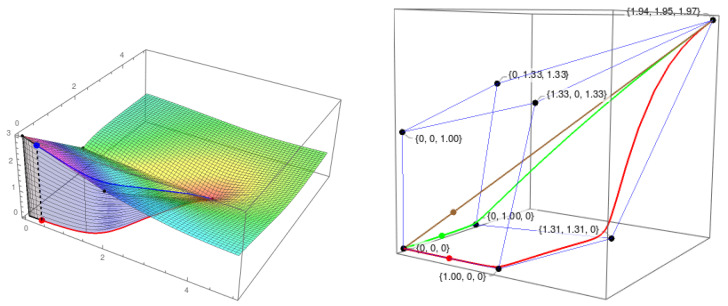
Metastability. Left: the IF of a two-node system is shown with the past (black shadow) and future (blue) trajectories of a point close to the *X*-axis. The trajectory finishes in the global stationary point, but it flows for a long time around the semi-stable stationary point in the *X*-axis. The right figure represents the phase space and the points in the IS of a three-node mechanism. The trajectories of three different points to the past and future are shown. The states are $\left(0.5,10^{-3},10^{-6}\right)$ for the red point and trajectories, $\left(10^{-3},0.5,10^{-3}\right)$ for the green and (0.3,0.3,0.3) for the brown one. Red and green solutions show two phenomena of metastability associated with different paths for saddle stationary points, one in the *X*-axis and the other in the *Y*-axis.

On the other hand, we have to deal with the concept of metastability [35,36,37], which tries to explain how closely the global solution visits small neighborhoods of semi-stable stationary points before reaching its final one. The way we measure metastability has to do with the spatial (geometrical closeness to a stationary point) and temporal (the time a global solution keeps inside a small neighborhood of a stationary point) characterization of a particular state when measuring its level of information (see Figure 4 for this kind of behavior associated with our model). We have the following result.

**Lemma** **2.** *Given level*$L_{k}$*in an IS, there exists*$\delta_{k}>0$*such that, for all*u∈A*such that*V(u)∈[r−k,r−k+1]*if the global solution*ξ:R→A*,*ξ(0)=u,*satisfies that there exists*$t^{*}>0$*such that*$S\left(t^{*}\right)u\in B\left(u_{j}^{*,k},\delta_{k}\right)$*, then*$S\left(t\right)u\notin B\left(u_{i}^{*,k},\delta_{k}\right),$*for all*$t\geq t^{*}$and$u_{i}^{*,k}\in L_{k}$*with*i≠j.

Indeed, every time a global solution associated with a particular u∈A visits one of these small neighborhoods of any of the stationary points at a particular level, a metastability phenomenon takes place, increasing the measure of information we will associate with this state.

## 4. Results

Now, we can introduce our approach to IIT for dynamical systems based on the notions of IS and IF. We consider substrates with a finite set of nodes P⊂N regulated by a system of ODEs:

(8) $$u_{i}^{\prime }\left(t\right)=u_{i}\left(t\right)\left(\alpha_{i}-u_{i}\left(t\right)+\sum_{j\in P,j\neq i}\gamma_{ij}u_{j}\left(t\right)\right)\;\mathrm{for}\;i\in P$$

with $\gamma_{ij}\geq 0$ for every i≠j, i,j∈P. It is equivalent to the formulation in (6), but making explicit all the relevant terms.

### 4.1. Intrinsic Existence

**Figure 5:**
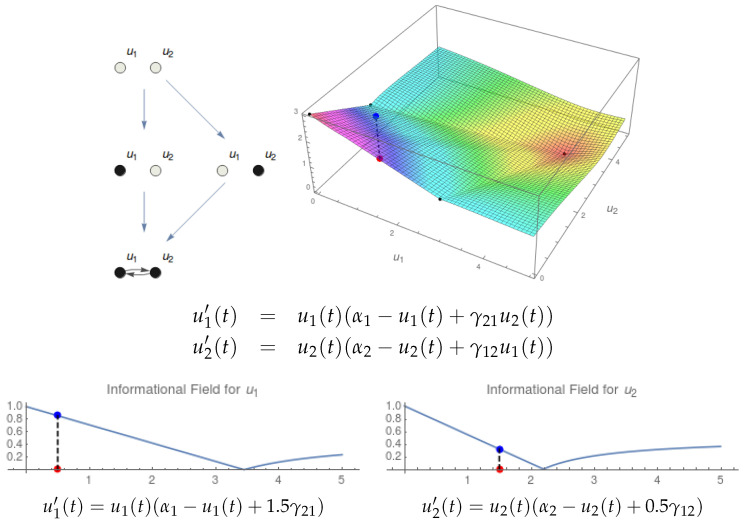
Existence and composition of a two-node mechanism $\left\{u_{1},u_{2}\right\}$ in a state. The parameters are: $\alpha_{1}=3$, $\alpha_{2}=2$, $\gamma_{12}=0.4$, $\gamma_{21}=0.3$. Top left: Informational structure with the four stable points of the system: (0,0), (3,0), (0,2), and (4.09,3.64). Top right: Informational field. The black points correspond to the points in the IS. The system is in state (0.5,1.5). The red point represents the state in the phase space, and the blue one is its projection in the IF. Bottom: Informational fields of the submechanisms $\left\{u_{1}\right\}$ (left) and $\left\{u_{2}\right\}$ (right) of the original mechanism. With one node only, the phase space has one coordinate, and the blue lines represent the corresponding informational fields. The state of the system in each of the submechanisms is shown. Observe that in the equation for the submechanism $\left\{u_{1}\right\}$ (left, below its IF), the variable $u_{2}\left(t\right)$ is replaced by the constant 1.5 with the value of $u_{2}$ in the given state of the system. It happens analogously in the case of the submechanism $\left\{u_{2}\right\}$.

The substrate defines a dynamical system on $R_{+}^{\left|P\right|}$. It has a global attractor A with a set of equilibrium points and their connections, and the dynamical system can be restricted to A such that each state has a well-defined past and future. Both the Informational Structure (IS, Section 3.3) and the Informational Field (IF, Section 3.4) exist. From the intrinsic perspective, the state of the system corresponds to a point in the IF (Figure 5). The key IIT notion for existence is the cause-effect power of the mechanism in a state. Now, the cause-effect power is given by the solutions of (8) when each $u_{i}\left(0\right)$ is the value of node *i* in the current state. Then, the cause power is given by the solution for t↦−∞, and the effect power is given by the solution for t↦∞. Figure 4 (left) shows an example of these solutions, which are associated with trajectories in the IF. In the example, the solution to the past (black lines in the phase space and IF) go to (0,0), and the solution to the future (red line in the phase space and blue line in the IF) goes to the global stable point. This way, we replace the TPM in IIT by dynamical notions derived from the system (8).

### 4.2. State-Dependent Composition

**Definition** **4.** *Let M be a substrate over P in state*$\hat{u}\in R^{\left|P\right|}$*. For every set of nodes*Q⊂P*, M is composed of the submechanism*$M_{Q}^{\hat{u}}$*with nodes Q and equations,*

(9) $$u_{i}^{\prime }\left(t\right)=u_{i}\left(t\right)\left(\alpha_{i}-u_{i}\left(t\right)+\sum_{j\in Q,j\neq i}\gamma_{ji}u_{j}\left(t\right)+\sum_{j\in P\setminus Q}\gamma_{ji}{\hat{u}}_{j}\right)\;for\;i\in Q$$

*Formally, the submechanism can be defined for every*$\hat{u}\in R^{\left|P\right|}$, but we will consider only states $\hat{u}\in A$*, the global attractor of the substrate.*

Figure 5 shows the existence and composition of a two-node mechanism over $\left\{u_{1},u_{2}\right\}$ in state (0.5,1.5). Observe the equations for $u_{1}^{\prime }\left(t\right)$ and $u_{2}^{\prime }\left(t\right)$ for the whole mechanism where both nodes are coupled. The Informational Structure (IS; top left) contains the solutions of this system and the Informational Field (IF; top right) the curvature of the phase space. The current state of the substrate is marked (blue point) in the IF. The system $\left\{u_{1},u_{2}\right\}$ is composed by two submechanisms $\left\{u_{1}\right\}$ and $\left\{u_{2}\right\}$ whose equations are shown below. Observe that in the equation for $u_{1}^{\prime }\left(t\right)$, the variable $u_{2}\left(t\right)$ does not appear; it is replaced by the constant 1.5, which is the value of $u_{2}$ in the current state. The same thing happens for $u_{2}^{\prime }\left(t\right)$. This produces that the phase space for each of the submechanisms has one dimension, and so, the IFs are one-dimensional curves.

### 4.3. Information

Any state $\hat{u}$ of the system lies in a particular point of the IF. Through this point, there exists a unique global solution ξ:R→X, $\xi \left(0\right)=\hat{u},$ of the system, which runs along the informational field for all past and future times.

We consider the positive and negative curves of this global solution given by $\Gamma^{+}={⋃}_{t\in R^{+}}\xi \left(t\right)$, $\Gamma^{-}={⋃}_{t\in R^{-}}\xi \left(t\right)$. On these curves, the IF is well defined and bounded. The cause information of a state in a system is the integral on the $\Gamma^{-}$ of the Lyapunov functional associated with the curve ξ(·), i.e.,

(10) $$ci\left(\hat{u}\right)=\int_{\Gamma^{-}}V\left(y\right)\;d\Gamma \left(y\right).$$

In the same way, the effect information of a state in a system is the integral on the $\Gamma^{+}$ of the Lyapunov functional associated with the curve ξ(·), i.e.,

(11) $$ei\left(\hat{u}\right)=\int_{\Gamma^{+}}V\left(y\right)\;d\Gamma \left(y\right).$$

Finally, the cause-effect information of a state in a system is:

(12) $$cei\left(\hat{u}\right)=\min\left\{ci\left(\hat{u}\right),ei\left(\hat{u}\right)\right\}$$

Observe that (11) and (10) are integrals of a scalar field over a curve, and they represent the area of the plane surface below the curve drawn by ξ(·).

**Figure 6:**
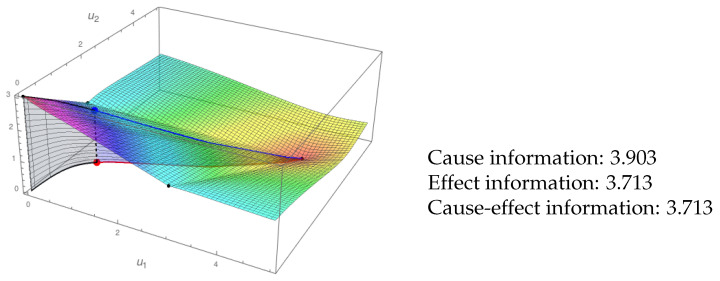
Cause-effect information of the system in Figure 5 also in state (0.5,1.5). The black area corresponds to the integral for the cause information, and the blue one is for effect information. The cause-effect information is the minimum of both values.

In the example of Figure 6, the red line is the trajectory (to the future) in the phase space, and the blue line is the projection of the red one over the scalar field (IF). The value of ei is the area of the (blue) surface between both lines. The calculation of ci is analogous (black area). In this way, we interpret the IIT requisite of a “difference that makes a difference” for information. Each point in the phase space has an associated future and past, and the amount of cause-effect information depends on the richness of the trajectories in the IF that the system can access in the current state. Contrary to our proposal in [11], we no longer need to define TPMs for the calculation of cei. Moreover, the cause-effect information can be calculated now for every point in the phase space A and not only for the finite set of points in the IS.

#### The Case of Stationary Points

As we have shown above, the skeleton of an informational field is made by its associated informational structure. Vertices of the informational structures are special states. Indeed, from a mathematical point of view, they are minimal invariants, so that the state is constant at all times; from an informational point of view, they are nodes from which a well-defined part of the informational field can be reached by their stable and unstable manifolds. Indeed, any stationary point is connected to the future (to the past) with a specific subset of vertices in lower (upper) informational levels. In this sense, they capture all the metastability phenomena of the system. This is the reason we have to define in a different way the quantity of information of these points:

(i) For the cause information (ci), we measure the total magnitudes of informational field along all possible paths joining the stationary point with the highest level stationary point for which there exists an indirect link between them. From all these paths, we take the one with the maximum value of the integral. If the stable manifold of the equilibrium is empty, i.e., the point has no connections with other equilibria to the past, we take ci=0.
(ii) For the effect information (ei), we measure the total magnitudes of the informational field along all possible paths joining the stationary point with the lowest level stationary point for which there exists an indirect link between them. From all these paths, we take the one with the maximum value of the integral. If the unstable manifold of the equilibrium is empty, i.e., the point has no connections with other equilibria to the future, we take ei=0.

**Figure 7:**
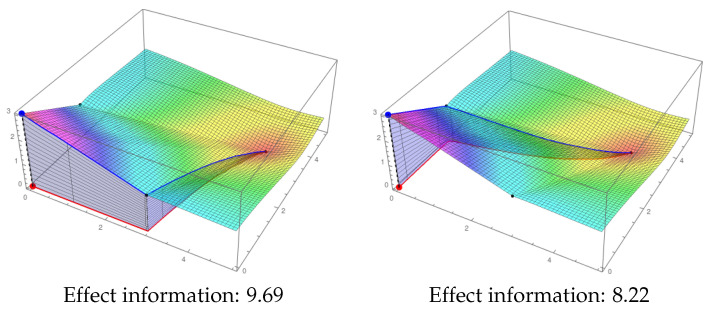
Effect information in the stable point (0,0). The IF represented in both figures corresponds to the two-node mechanism in Figure 5. In the left side, the path that passes through the point (3,0) is considered. The effect information that corresponds to that path (blue area) is 9.69. In the right side, the path passes through (0,2), and the value of the blue area is 8.22. Then, the effect information of this mechanism in the state (0,0) is 9.69 as it is the greatest integral of the possible paths moving from (0,0) to the global stable point (4.09,3.64).

As usual, the level of information of stationary points is given by the minimum of ci and ei. An example of the computation of ei for the stable point (0,0) is presented in Figure 7.

### 4.4. Integration

We first start by defining how a partition of a substrate is done.

**Definition** **5.** *Let M be a substrate over P and a set* Q⊂P *(with* Q≠P *and* Q≠∅ *). Given the state* $\hat{u}\in R^{N}$ *, the partition* $M_{PART(Q)}^{\hat{u}}$ *over P is a substrate (also over P) whose equations are those in* $M_{Q}^{\hat{u}}$ *and* $M_{P\setminus Q}^{\hat{u}}$ *(see Definition 4).*

**Figure 8:**
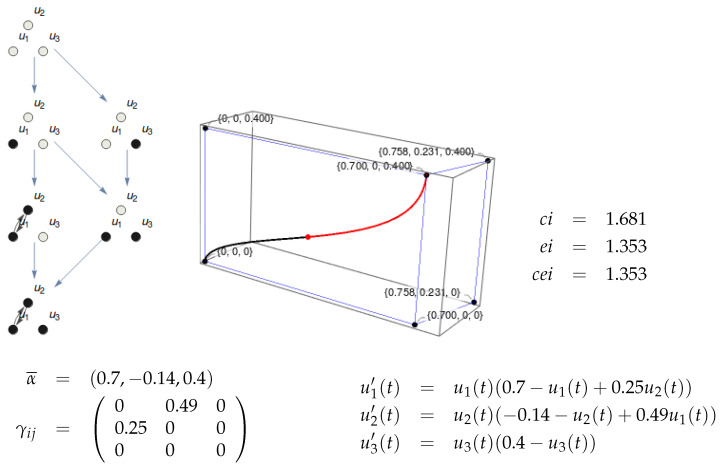
Existence and information of a three-node mechanism in state (0.36,0,0.16). Top Left: Informational structure of the system corresponding to the $\bar{\alpha }$ and $\gamma_{ij}$ parameters below. Top right: Phase space with the nodes of the IS in black. The red point is the state of the system, and the black and red lines are the trajectories in the phase space for ci and ei, respectively. The IF cannot be represented as it lies in $R^{4}$, but the value of the Lyapunov function in each point can be calculated (it is 3.47 for the current state). Bottom: Parameters of the mechanism and differential equations of the system. It can be observed that $u_{1}$ and $u_{2}$ have a mutual dependence, while $u_{3}$ is not related to the other variables. As a consequence, the system has no integration as the partition $\left\{u_{1},u_{2}\right\}/\left\{u_{3}\right\}$ produces the same IS and IF as the original mechanism.

**Figure 9:**
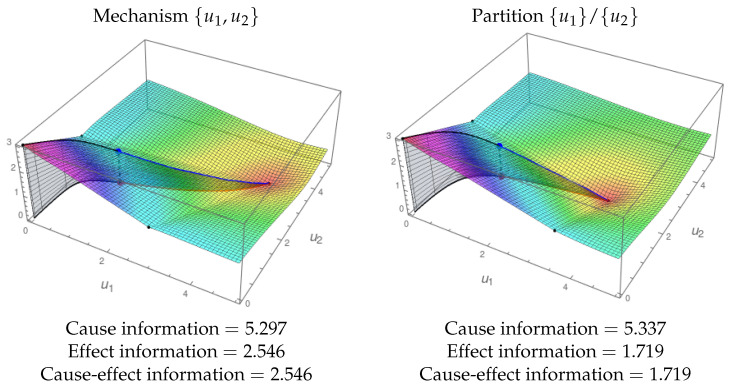
Cause-effect information in a mechanism $\left\{u_{1},u_{2}\right\}$ and the partition $\left\{u_{1}\right\}/\left\{u_{2}\right\}$. In both cases, the state is (1,2). The left IF corresponds to the mechanism in Figure 5. There is no integration as the IS of the partition has the same structure. The equations to build the IS and IF of the partition are those depicted in Figure 5 (bottom), but one and two are used instead of 0.5 and 1.5 as the constant values for the variables outside each subsystem.

Given a substrate in a particular state, it generates an Informational Structure (IS) and an Informational Field (IF). When we make a partition (Definition 5) of the substrate, we get an IF that may be topologically equivalent to the original one. The substrate is integrated in a particular state when this is not the case. Figure 8 represents a trivial case of a substrate with no integration because one of its nodes ($u_{3}$) is isolated. The IS and IF are trivially identical to those of its partition $\left\{u_{1},u_{2}\right\}/\left\{u_{3}\right\}$. Figure 9 shows a more interesting example of a partition of a substrate with two nodes where the topology of the resulting IF is identical to the original one. Though the ci and ei are different in the original system and the partition, there is no integration.

We stress that there are two possible choices on how to define if there exists integrated information in the system. The way we measure the quantity of information is very accurate due to our continuous framework. Thus, as in the example in Figure 9, the partitioned system leads to the same IS even if the value of φ is nonzero. We could say that there is integration in such a case, even though ISs are the same and IFs stay topologically equivalent. Following this definition, integration, perhaps with some small values of φ, would be a generic property present in almost all systems that are not disconnected. We think we should take it into account in order to distinguish different, but close enough experiences. However, we choose another stronger notion of integration, requiring that the structure of the underlying IS changes after partition. In that case, the difference between the original substrate and its partition is not only of a quantitative, but also of a qualitative nature. This way, to obtain integration, we require not only that the value of φ is nonzero for all partitions, but also that the partitions lead to the change of the IS’s structure. We remark about the fact that the intrinsic structure of the IS could not be recovered from any of its partitions (which could reflect that a conscious experience cannot be reduced, in a qualitative, and not only quantitative way, to its components).

Then, the substrate *M* has integration for state $\hat{u}$ if its associated IF cannot be reached (as a topological conjugation) from any partition of the substrate. The measure of integrated information φ of the system is calculated as the minimum of $\varphi_{\mathrm{cause}}$ and $\varphi_{\mathrm{effect}}$. The value of $\varphi_{\mathrm{cause}}$ is given by:

$$\varphi_{\mathrm{cause}}\left(\hat{u}\right)=|ci\left(\hat{u}\right)-ci^{MIP(\mathrm{cause})}\left(\hat{u}\right)|,$$

where $ci(\hat{u})$ is the cause information of *M* in state $\hat{u}$ and $ci^{MIP(\mathrm{cause})}\left(\hat{u}\right)$ is the cause information of the partition MIP(cause) in the same state $\hat{u}$. By MIP(cause), the minimum cause information partition, we understand from such a partition of *M* that its cause information is closest to $ci(\hat{u})$. The value of $\varphi_{\mathrm{effect}}$ is calculated in an analogous way, but for effect information.

**Figure 10:**
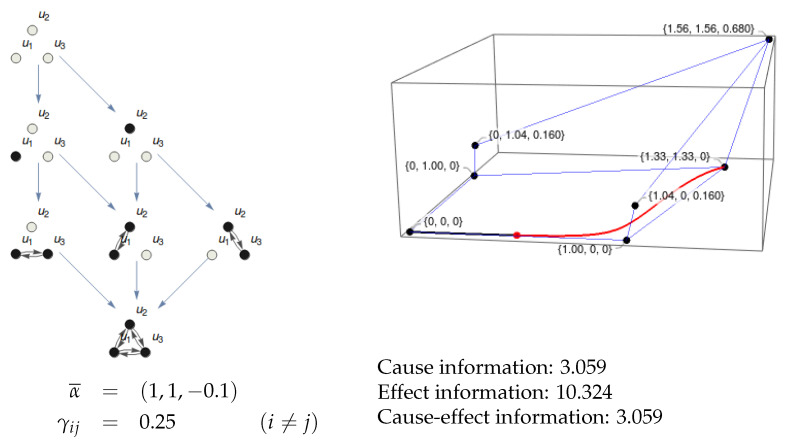
Existence and information of a three-node substrate. The IS is shown at the left. It corresponds to a three-node substrate with $\alpha_{1}=\alpha_{2}=1$, $\alpha_{3}=-0.1$, and $\gamma_{ij}=0.25$ for all connections between different nodes. At the right, the nodes of the IS are shown in the phase space together with the past (black line) and future (red line) solutions for the state (0.5,0.01,0). The projections in the IF cannot be shown as the IF lies in $R^{4}$, but the integrals can be calculated. The values for ci, ei, and cei are shown.

**Figure 11:**
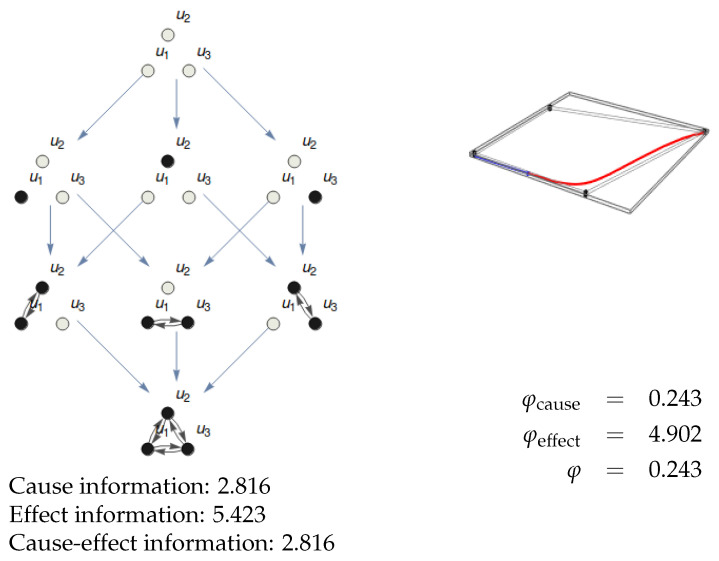
Integration of the system in Figure 10. The informational structure of the partition $\left\{u_{1},u_{2}\right\}/\left\{u_{3}\right\}$ is shown in the left. It is the one with both most similar ci and ei values to those of the original system. The integration φ is the minimum of $\varphi_{\mathrm{cause}}$ and $\varphi_{\mathrm{effect}}$, which are the (absolute value of the) differences between cause information and effect information in the original system and the partition.

Figure 10 shows an example of a substrate with three nodes. The IF cannot be shown as it lies in $R^{4}$, but the integrals for ci and ei can be calculated. The integration for this system in state (0.5,0.01,0) is shown in Figure 11. The MIP is $\left\{u_{1},u_{2}\right\}/\left\{u_{3}\right\}$ both for the cause and the effect. Observe that the topology of the resulting IS (eight nodes) is different to that of the original system (seven nodes). Therefore, the integration φ can be calculated as the minimum of $\varphi_{\mathrm{cause}}$ and $\varphi_{\mathrm{effect}}$.

We remark that, as the calculation of φ involves taking the minimum of $\varphi_{\mathrm{cause}}$ and $\varphi_{\mathrm{effect}}$, the states for which either of these values is low, which could be points located near the globally-stable or globally-unstable equilibria of the system, will not lead to high φ. On the other hand, the points which have the best balance between ci and ei can potentially lead to a higher value of φ. We note, however, that the values of ci and ei do not depend merely on the distance between the current state and the equilibrium in the phase space, but on the trajectory and the value of the IF. Hence, for example, due to metastability or due to the presence of the imaginary part of the eigenvalue for a limit equilibrium, it may happen that the distance between the given state and the corresponding limit point is low, but the associated value of ci or ei is high. Moreover, this behavior can be highly dependent on the particular system of equations chosen on the substrate.

### 4.5. Exclusion

The exclusion postulate, in our framework, aims to select the subset of nodes in the substrate that produces the maximum of integrated information (small) φ. Given a substrate *M* over *P* in state $\hat{u}$, it may happen that the φ value for *M* is lower than that of a submechanism $M_{Q}^{\hat{u}}$ for some Q⊂P (see Definition 4). Then, the subset S⊂P maximizing φ gives the submechanism $M_{S}^{\hat{u}}$ producing the conscious experience.

**Figure 12:**
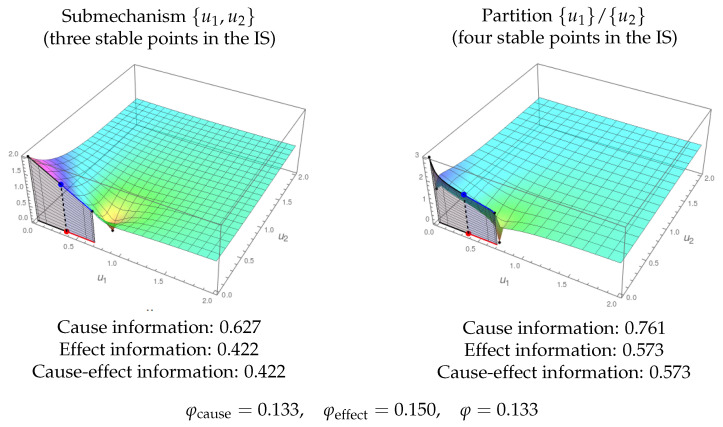
Integration and exclusion. Left: IF corresponding to the submechanism given by the nodes $\left\{u_{1},u_{2}\right\}$ of the substrate in Figure 8 (with nodes $\left\{u_{1},u_{2},u_{3}\right\}$). It is in state (0.36,0) (the original state was (0.36,0,0.16)). Cause and effect information is shown. Right: IF of the partition $\left\{u_{1}\right\}/\left\{u_{2}\right\}$ in the same sate. Given that the structure of the ISs is different for $\left\{u_{1},u_{2}\right\}$ (three nodes) and $\left\{u_{1}\right\}/\left\{u_{2}\right\}$ (four nodes), the substrate is integrating information. Recall that the original substrate does not integrate information as $u_{3}$ is disconnected from $u_{1}$ and $u_{2}$ (Figure 8). As this submechanism from $\left\{u_{1},u_{2}\right\}$ is the one integrating more information, the exclusion postulate determines that this is the one contributing to the conscious experience.

Figure 12 is an example of the integration and exclusion postulates. It shows integration because the only partition of the substrate $\left\{u_{1},u_{2}\right\}$ that produces a topologically different informational field is $\left\{u_{1}\right\}/\left\{u_{2}\right\}$, so that we can measure the level of integrated information as the difference between the information of $\left\{u_{1},u_{2}\right\}$ and $\left\{u_{1}\right\}/\left\{u_{2}\right\}$. The exclusion postulate is illustrated because $\left\{u_{1},u_{2}\right\}$ is the only submechanism of the whole substrate $\left\{u_{1},u_{2},u_{3}\right\}$ in Figure 8 that integrates information. Therefore, the submechanism producing the conscious experience is given by $\left\{u_{1},u_{2}\right\}$.

**Figure 13:**
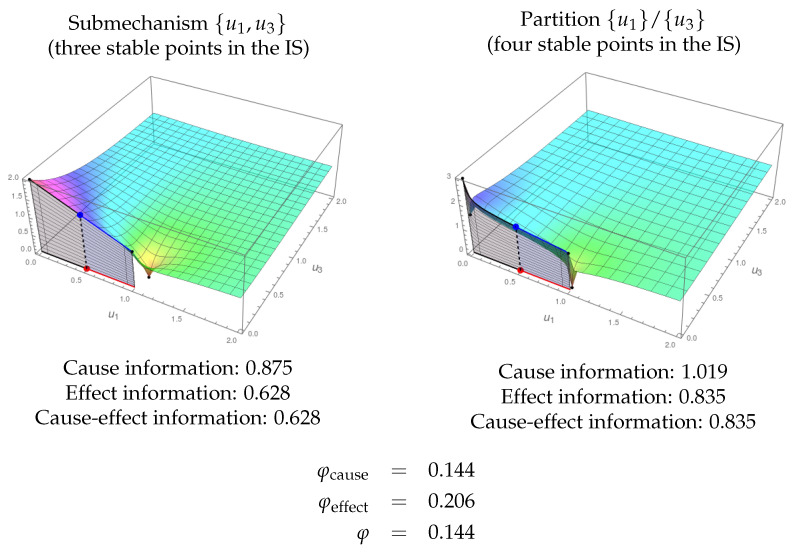
Exclusion. The submechanism given by $\left\{u_{1},u_{3}\right\}$ of the system in Figure 10 in state (0.5,0) has integrated information, φ=0.144. In the figure, both the IF for the submechanism $\left\{u_{1},u_{3}\right\}$ (left) and for the partition $\left\{u_{1}\right\}/\left\{u_{3}\right\}$ (right) are shown. However, as the integrated information is lower than that of the complete system (Figure 11, φ=0.243), the exclusion postulate chooses the whole substrate $\left\{u_{1},u_{2},u_{3}\right\}$ as the one producing more integrated information.

Figure 13 shows another example of the exclusion postulate. In this case, the submechanism $\left\{u_{1},u_{3}\right\}$ of a whole system $\left\{u_{1},u_{2},u_{3}\right\}$ integrates information, but the φ value for $\left\{u_{1},u_{3}\right\}$ is lower than that for $\left\{u_{1},u_{2},u_{3}\right\}$. Therefore, in this case, the exclusion postulate selects the whole substrate as the system producing the conscious experience.

## 5. Discussion

The main contribution of this paper is the introduction of informational structures and informational fields as a prototype for a continuous description of the postulates of IIT. For a given substrate, the system of ODEs and the high complexity and richness of its IF provide more powerful possibilities than those supplied by exploring the behavior and dynamics of just the substrate itself. IFs and ISs exist in a curved well-organized phase space. The qualitative and quantitative properties of any state follow from an abstract framework based on the IS and IF.

We have gone one step further from our previous approach in [11]. In that paper, the core concept was IS, for which we defined (a finite) set of states associated with the stationary points. Thus, a TPM was obtained with the probability distributions associated with the finite set of stationary points in the IS. In this way, we moved from the IS to a discrete framework, where we were able to mimic the IIT approach in [10] partially. In the present paper, the perspective was totally different (see Results), so that, with the aim to proceed in a completely continuous fashion, the description of IIT postulates was directly developed by taking advantage of some of the main concepts and results from the recent theory of dynamical systems on complex networks. The Lyapunov functional and its associated informational field are now the core concepts allowing for a continuous description for the level of integrated information of any state. Thus, a state in the IF is shown to have cause-effect power, similarly as the finite number of discrete states in IIT, but the way to measure their level of integrated information is totally different. While IIT in [10] uses the EMD between probability distributions to measure intrinsic information, our approach to measure the cause-effect power of a state is based on the mathematical analysis associated with integrals of a scalar field along trajectories. However, we are still far from coping with all IIT 3.0 requirements. For clarity, we have just presented a concept of integrated information referring to the whole substrate, so that we lose the compositional way of measuring information in IIT 3.0 and, so, the way it computes (small) φ. Thus, what we have shown is just a first approach to φ (and not to (big) Φ). We do not think it is a limitation of our approach, and we could have done it in this way, but probably loosing some clarity in the presentation. In any case, it deserves to be developed in further work.

We also dealt with the concept of metastability [35,38,39,40], showing that this is a strong concept that enriches the level of information of any state. In our approach, metastability appears when a solution, even converging to a global asymptotically-stationary point, spends a long time around semi-stable solutions. Observe that metastability is one of the properties that has been suggested to contribute to consciousness in the resting state [40]. Moreover, metastability makes a solution be driven close to the limits of the informational structure for which bifurcation phenomena [1] may happen, another important issue related to critical behavior in brain dynamics [1,37,40,41].

There is still a long way until these concepts can be formulated in their final form, as was done in the current development of IIT 3.0. Some of our perspectives for further research are:

(a) As already pointed, a necessary interesting research direction is performing further comparison of the features of our approach in relation to IIT. Introduction of the compositional way to measure the information will thus enable the extension of the proposed approach to calculate not only the equivalent of “small phi” φ, but also of “big Phi” Φ.
(b) For global brain dynamics, the substrate describing structural connectivity is usually given by known parcellations of the brain [42]. Multilevel networks [43,44,45,46] and the relation with ISs and IFs have to be introduced for a development of this approach in order to deal with real data.
(c) We have used in the examples a Lotka–Volterra system of a cooperative nature. This produces that the nodes in the IS are hierarchically organized stationary points. From a theoretical point of view, other possible models for brain dynamics could be used. The fundamental theorem of dynamical systems [19,23] guarantees, under some conditions, the existence of ISs. However, the nodes in these generalized structures are not always stationary points, but minimal invariant recurrent sets, containing, for instance, homoclinic structures or even chaotic behavior.
(d) There exists a huge literature on the dependence between the topology of underlying graphs and their associated dynamics [44,45,47,48,49,50,51,52]. To our knowledge, it is still not totally clear what this dependence refers to, sometimes suggesting that the dynamics and observed functionality are strongly determined by the topological structure of the graphs. The relation between the topology of the substrate and the one of the informational field is then a very important research area.
(e) The effective calculation of a Lyapunov function is not an easy task, and we have used a general way to do it as done in [9,17]. It is an approach general enough to deal with arbitrary *n*-dimensional substrates, for which the existence of a Lyapunov function is well settled.
(f) A promising future work is related to the possibility of considering time-dependent parameters in (5). Then, a continuous flow of ISs and IFs is generated. Our abstract approach can be extended, in a sensible and natural way, for this class of non-autonomous systems. In particular, we have adopted this perspective in [34], where we have described the ISs of eighteen people in the resting state and N3 deep sleep. The results showed a significative higher variability in the lowest stationary point for awake people with respect to unconsciousness states. We think these results could open the door for further studies of ISs and IFs for real brain data. In particular the fractal dimension of brain data using transcranial magnetic stimulation was analyzed in [53]. Although not directly related to ISs, it is again a dynamical system framework that supplies a characterization of conscious and different unconscious states.

## References

[1] Deco G., Jirsa V. (2012). Ongoing cortical activity at rest: Criticality, multistability, and ghost attractors. J. Neurosci..

[2] Ghosh A., Rho Y., McIntosh A., Kotter R., Jirsa V. (2008). Cortical network dynamics with time delays reveals functional connectivity in the resting brain. Cogn. Neurodyn..

[3] Deco G., Tononi G., Boly M., Kringelbach M. (2015). Rethinking segregation and integration: Contributions of whole-brain modelling. Nat. Rev. Neurosci..

[4] Honey C., Kotter R., Breakspear M., Sporns O. (2007). Network structure of cerebral cortex shapes functional connectivity on multiple time scales. Proc. Natl. Acad. Sci. USA.

[5] Rabinovich M., Varona P., Tristan I., Afraimovich V. (2014). Chunking dynamics: Heteroclinics in mind. Front. Comput. Neurosci..

[6] Ponce-Alvarez A., Deco G., Hagmann P., Romani G., Mantini D., Corbetta M. (2015). Resting-State Temporal Synchronization Networks Emerge from Connectivity Topology and Heterogeneity. PLoS Comput. Biol..

[7] Deco G., Kringelbach M. (2016). Metastability and Coherence: Extending the Communication through Coherence Hypothesis Using A Whole-Brain Computational Perspective. Trends Neurosci..

[8] Guerrero G., Langa J.A., Suárez A. (2016). Attracting complex networks. Complex Networks and Dynamics.

[9] Guerrero G., Langa J.A., Suárez A. (2017). Architecture of attractor determines dynamics on mutualistic complex networks. Nonlinear Anal. Real World Appl..

[10] Oizumi M., Albantakis L., Tononi G. (2014). From the Phenomenology to the Mechanisms of Consciousness: Integrated Information Theory 3.0. PLoS Comput. Biol..

[11] Esteban F.J., Galadí J.A., Langa J.A., Portillo J.R., Soler-Toscano F. (2018). Informational structures: A dynamical system approach for integrated information. PLoS Comput. Biol..

[12] Babin A.V., Vishik M. (1938). Regular attractors of semigroups and evolution equations. Math. Pures Appl..

[13] Hale J.K. (1988). Asymptotic Behavior of Dissipative Systems.

[14] Henry D.B. (1981). Geometric Theory of Semilinear Parabolic Equations.

[15] Ladyzhenskaya O.A. (1991). Attractors for Semigroups and Evolution Equations.

[16] Temam R. (1997). Infinite Dimensional Dynamical Systems in Mechanics and Physics.

[17] Aragão-Costa E.R., Caraballo T., Carvalho A.N., Langa J.A. (2011). Stability of gradient semigroups under perturbations. Nonlinearity.

[18] Carvalho A., Langa J., Robinson J. (2012). Attractors for Infinite-Dimensional Non-Autonomous Dynamical Systems.

[19] Conley C. (1978). Isolated Invariant Sets and the Morse Index.

[20] Hurley M. (1995). Chain recurrence, semiflows, and gradients. J. Dyn. Differ. Equ..

[21] Strogatz S.H. (2015). Nonlinear Dynamics and Chaos.

[22] Wiggins S. (2003). Introduction to Applied Nonlinear Dynamical Systems and Chaos.

[23] Norton D.E. (1995). The fundamental theorem of dynamical systems. Comment. Math. Univ. Carol..

[24] Robinson J.C. (2001). Infinite-Dimensional Dynamical Systems.

[25] Patrão M., San Martin L.A.B. (2007). Semiflows on topological spaces: Chain transitivity and semigroups. J. Dyn. Differ. Equ..

[26] Rybakowski K.P. (1987). The Homotopy Index and Partial Differential Equations.

[27] Aragão-Costa E.R., Caraballo T., Carvalho A.N., Langa J.A. (2012). Continuity of Lyapunov functions and of energy level for a generalized gradient semigroup. Topol. Methods Nonlinear Anal..

[28] Afraimovich V.S., Moses G., Young T. (2016). Two-dimensional heteroclinic attractor in the generalized Lotka-Volterra system. Nonlinearity.

[29] Afraimovich V.S., Zhigulin V.P., Rabinovich M.I. (2004). On the origin of reproducible sequential activity in neural circuits. Chaos.

[30] Muezzinoglu M.K., Tristan I., Huerta R., Afraimovich V.S., Rabinovich M.I. (2010). Transients versus attractors in complex networks. Int. J. Bifurc. Chaos Appl. Sci. Eng..

[31] Yi Z. (2010). Foundations of Implementing the Competitive Layer Model by Lotka-Volterra Recurrent Neural Networks. IEEE Trans. Neural Netw..

[32] Takeuchi Y. (1996). Global Dynamical Properties of Lotka-Volterra Systems.

[33] Takeuchi Y., Adachi N. (1980). The existence of globally stable equilibria of ecosystems of the generalized Volterra type. J. Math. Biol..

[34] Silva Pereira S., Galadí J., Langa J., Gayte I., Suárez A., Tagliazucchi E., Laufs H., Deco G. (2019). Informational Structures and Underlying Energy Landscapes: Characterizing Brain States.

[35] Fusco G., Hale J.K. (1989). Slow-motion manifolds, dormant instability, and singular perturbations. J. Dyn. Differ. Equ..

[36] Tognili E., Scott Kelso J. (2014). The Metastable Brain. Neuron.

[37] Werner G. (2007). Metastability, criticality and phase transitions in brain and its models. Biosystems.

[38] Afraimovich V.S., Muezzinoglu M.K., Rabinovich M.I. (2010). Metastability and transients in brain dynamics: Problems and rigorous results. Long-Range Interactions, Stochasticity and Fractional Dynamics.

[39] Hansen E., Battaglia D., Spiegler A., Deco G., Jirsa V. (2015). Functional connectivity dynamics: Modeling the switching behavior of the resting state. NeuroImage.

[40] Deco G., Kringelbach M.L., Jirsa V., Ritter P. (2017). The dynamics of resting fluctuations in the brain: Metastability and its dynamical cortical core. Sci. Rep..

[41] Chialvo D.R. (2014). Critical Brain Dynamics at Large Scale. Criticality in Neural Systems.

[42] Sporns O. (2010). Networks of the Brain.

[43] Leergaard T., Hilgetag C., Sporns O. (2012). Mapping the Connectome: Multi-Level Analysis of Brain Connectivity. Front. NeuroInform..

[44] Bullmore E., Sporns O. (2009). Complex brain networks: Graph theoretical analysis of structural and functional systems. Nat. Rev. Neurosci..

[45] Chu S.H., Parhi K.K., Lenglet C. (2018). Function-specific and Enhanced Brain Structural Connectivity Mapping via Joint Modeling of Diffusion and Functional MRI. Sci. Rep..

[46] Danziger M.M., Bonamassa I., Boccaletti S., Havlin S. (2018). Dynamic interdependence and competition in multilayer networks. Nat. Phys..

[47] Park H.J., Friston K. (2013). Structural and Functional Brain Networks: From Connections to Cognition. Science.

[48] Deco G., Senden M., Jirsa V. (2012). How anatomy shapes dynamics: A semi-analytical study of the brain at rest by a simple spin model. Front. Comput. Neurosci..

[49] Stam C. (2010). Characterization of anatomical and functional connectivity in the brain: A complex networks perspective. Int. J. Psychophysiol..

[50] Bascompte J., Jordano P., Melián C.J., Olesen J.M. (2003). The nested assembly of plant–animal mutualistic networks. Proc. Natl. Acad. Sci. USA.

[51] Bascompte J., Jordano P. (2007). Plant-Animal Mutualistic Networks: The Architecture of Biodiversity. Annu. Rev. Ecol. Evol. Syst..

[52] Bastolla U., Fortuna M.A., Pascual-García A., Ferrera A., Luque B., Bascompte J. (2009). The architecture of mutualistic networks minimizes competition and increases biodiversity. Nature.

[53] Ruiz de Miras J., Soler-Toscano F., Iglesias-Parro S., Ibánez-Molina A., Casali A., Laureys S., Massimini M., Esteban F., Navas J., Langa J. (2019). Fractal Dimension Analysis of States of Consciousness and Unconsciousness Using Transcranial Magnetic Stimulation. Comput. Methods Programs Biomed..

